# Proximal Gamma-Ray Spectroscopy to Predict Soil Properties Using Windows and Full-Spectrum Analysis Methods

**DOI:** 10.3390/s131216263

**Published:** 2013-11-27

**Authors:** Hafiz Sultan Mahmood, Willem B. Hoogmoed, Eldert J. van Henten

**Affiliations:** 1 Farm Technology Group, Wageningen University, PO Box 317, 6700 AH Wageningen, The Netherlands; E-Mails: willem.hoogmoed@wur.nl (W.B.H.); eldert.vanhenten@wur.nl (E.J.H.); 2 Agricultural and Biological Engineering Institute, National Agricultural Research Centre, Park Road, PO Box 45500, Islamabad, Pakistan

**Keywords:** proximal soil sensing, gamma-ray spectroscopy, energy windows, full-spectrum analysis, prediction of soil properties

## Abstract

Fine-scale spatial information on soil properties is needed to successfully implement precision agriculture. Proximal gamma-ray spectroscopy has recently emerged as a promising tool to collect fine-scale soil information. The objective of this study was to evaluate a proximal gamma-ray spectrometer to predict several soil properties using energy-windows and full-spectrum analysis methods in two differently managed sandy loam fields: conventional and organic. In the conventional field, both methods predicted clay, pH and total nitrogen with a good accuracy (R^2^ ≥ 0.56) in the top 0–15 cm soil depth, whereas in the organic field, only clay content was predicted with such accuracy. The highest prediction accuracy was found for total nitrogen (R^2^ = 0.75) in the conventional field in the energy-windows method. Predictions were better in the top 0–15 cm soil depths than in the 15–30 cm soil depths for individual and combined fields. This implies that gamma-ray spectroscopy can generally benefit soil characterisation for annual crops where the condition of the seedbed is important. Small differences in soil structure (conventional *vs.* organic) cannot be determined. As for the methodology, we conclude that the energy-windows method can establish relations between radionuclide data and soil properties as accurate as the full-spectrum analysis method.

## 1. Introduction

Characterisation of spatial variability in soil properties is crucial for farmers to reduce the risk of crop failure, to improve the efficiency of decision making and to benefit in both the economic and environmental sense [1]. Collection of fine-scale information on soil properties, using conventional soil sampling and laboratory analyses, is time consuming and expensive. More efficient methods to obtain this information are essential for soil monitoring, modelling and precision agriculture [2].

Proximal soil sensors in precision agriculture have the capability to provide soil information with high spatial resolution and to explain variations in soil properties [3]. These soil sensing methods are subject to less interfering factors, such as clouds and vegetation cover and are advantageous over aerial and satellite remote sensing methods [4]. Using proximal sensors, therefore, less ambiguous relations can be established between sensor-output and soil properties [5]. Gamma-ray spectroscopy, also known as radiometrics, is one of the ground-based proximal soil sensing methods that can provide information on soil properties at a high spatial resolution.

Gamma rays are quanta or photons of high energy and short-wavelength electromagnetic radiation emitted from naturally occurring isotopes [6]. Radioactive isotopes of elements that emit gamma radiation are called radionuclides. Many radionuclides occur naturally, but only potassium (K) and the decay series of uranium (U) and thorium (Th) produce gamma rays of sufficient energy and intensity to be measured with gamma-ray spectroscopy. These radionuclides are present in soils and rocks in the form of ^40^K, ^238^U and ^232^Th isotopes in varying amounts. Other man-made or human induced radionuclides are also present in soils, such as ^137^Cs (a radioisotope of caesium) that has been deposited on soils due to nuclear tests, warfare and accidents like Chernobyl [7].

Radiation not originating from the Earth's surface is regarded as background. The main sources of background radiation are atmospheric radon (^222^Rn), cosmic sources and instrumental background. A number of factors attenuate gamma-ray emission. In soils, water content and bulk density are the major factors that attenuate gamma-ray emission [8].

The abundance and distribution of radionuclides reflect geomorphic and weathering processes [9,10]. Sandy soils with leached profiles, are readily recognised by a low gamma-ray count rate [11]. In clayey soils, ^232^Th can adsorb onto clays and hence clay content can be mapped from ^232^Th concentration [12]. Potassium feldspars occur in granites. Freshly weathered granite with a shallow soil profile has a high ^40^K count rate [11,13]. Ferruginous materials or gravels from a deeply weathered profile are rich in ^232^Th and ^238^U counts [9,11]. The concentration of ^40^K, ^232^Th and ^238^U contents in soils and rocks generally increases with increasing silica content [13]. Soil texture is more likely to contribute directly to the radiometric data than the other soil properties, such as organic carbon or pH [14]. Once a relationship is established with soil texture, many other indirect relationships between soil properties and radiometric data are apparent [15].

Gamma-ray spectroscopy is a relatively new approach in characterising soil properties for arable farming and the focus has been to evaluate the technology in a soil mapping framework. Proximal gamma-ray spectroscopy may provide significant advantages when compared to other proximal soil sensing methods, such as visible-near infrared spectroscopy and electromagnetic induction (EMI) methods. It is a non-invasive and non-destructive method for topsoil sensing and mapping. Using gamma-ray spectroscopy, soil variables can be mapped at a high spatial resolution [16], dense vegetation can only reduce elemental readings by 15% [17], gamma rays can be related with clay mineralogy and soil chemistry [9] and the concentration of radionuclides can be related with soil properties using simple correlation method. Furthermore, unlike EMI sensors, metal objects do not attenuate gamma rays while soil measurement. Several authors have identified relationships between airborne gamma-ray data and soil properties. Most of them established correlations between soil properties and energy-windows (EWs) of radionuclides, such as ^40^K (EW_K_), ^238^U (EW_U_) and ^232^Th (EW_Th_). Highly weathered residuum was distinguished from fresh material of granitic outcrops and identified soil parent materials using EWs of airborne gamma-ray spectroscopy [11]. Total phosphorus content (P) was predicted (R^2^ = 0.78) with EW_K_ when combined with several parameters of a digital elevation model [18]. Clay content was predicted (R^2^ = 0.68) with EW_Th_ using linear regression in highly weathered soils [19]. In a later study, the EWs of airborne gamma-ray spectra were related with clay content and plant available potassium (available-K) in highly weathered and varying textured farmland [20]. Airborne gamma-ray studies in young soils were reported from Wales and England [21], where good correlations of EW_K_ and EW_Th_ were found with soil parent materials and soil texture.

Although airborne gamma-ray spectroscopy has been used to find relationships with soil properties, it cannot distinguish small variations in soil properties within a field. Higher altitude causes more attenuation, lowers the spatial resolution and thus lowers the intensity of signal, which is reduced to half above 121 m in air [11].

Proximal gamma-ray spectrometers gained interest since the last decade for soil property mapping with spectral EWs of radionuclides. A good correlation (R^2^ = 0.93) was found between plant available-K and EW_K_ on a large farm in Australia [15]. The EW_K_ was further found to be related with clay content, pH, iron (Fe), P and organic carbon.

Attempts were also made to relate gamma-ray data with soil properties using full-spectrum analysis (FSA) methods. The data of a proximal gamma-ray spectrometer were analysed using an FSA method based on partial least squares regression (PLSR) and found robust predictions for clay, sand and Fe content (R^2^ ≥ 0.63) in the top 0–15 cm soil depth [22]. An FSA method was proposed [23] to relate radiometric data with environmental attributes. This method has been reported to relate soil texture (clay content), organic matter and soil nutrients with variable success [24]. Clay content was determined using gamma-ray spectroscopy when combined with this FSA method in three marine districts in the Netherlands at field, regional and district levels [25].

It is expected that gamma-ray spectroscopy can enhance spatial resolution of soil data at the field scale when analysed with either the EWs or the FSA methods. Although a certain amount of relevant information may be lost in the EWs method, this serves as a simple and reference method to relate radiometric data with soil properties. A proximal gamma-ray spectrometer commonly used in the Netherlands, the Mole (the Mole is a gamma-ray spectrometer developed and commercially used by The Soil Company, Groningen, The Netherlands), was developed for the FSA method [24] and to the best of our knowledge no attempts have been made to evaluate it with the simpler EWs method.

The objective of this study was to evaluate a proximal/ground-based gamma-ray spectrometer to find quantitative relationships between radiometric data and soil properties, such as texture, EC, pH, total organic carbon and total nitrogen, in two closely located sandy loam fields in The Netherlands. Our further intent was to compare the abilities of two data analysis methods: the EWs and the FSA, to predict soil properties using the radiometric data acquired by this spectrometer.

## 2. Experimental Section

### 2.1. Study Fields

This study was conducted at the experimental farm of Wageningen University, the “Broekemahoeve”, near Lelystad (52°32′35.67″N, 5°34′26.50″E), in the Flevoland province of The Netherlands. Total study area was about 4 ha, comprising a conventionally managed and an organically managed field, approximately 100 m apart. In the conventional field, fertilisers and chemicals were applied for nutrients and to control insects, diseases and weeds. The organic field was managed without chemicals. Soil texture of the fields was sandy loam with varying amount of seashells. Fields were under a wheat crop and measurements were carried out after the harvest in the year 2010.

### 2.2. Acquisition of Gamma-Ray Data

The gamma-ray data were acquired using a portable passive gamma-ray spectrometer, the Mole, equipped with a CsI (Tl) scintillation crystal detector. The CsI(Tl) crystal (70 × 150 mm) is coupled with a photomultiplier unit and a multichannel analyser (MCA) system to acquire real-time gamma-ray spectra. The MCA system consists of 256 energy channels between 0 and 3.0 MeV. The spectrometer can be mounted on a tractor, car, and quad bike or can even be used manually. In this study, the spectrometer was moved in the field at about 1.2 m·s^−1^ mounted on a wheel barrow at about 30 cm height. The field of view of the spectrometer at this height was about 3 m radius. In each field, eight rows were selected for data collection, which were about 10 m apart along the length of the fields. The radiometric data were collected at 1 Hz frequency from about 4,000 points from both fields together with the associated GPS locations with a measurement accuracy of 1 m. These data were logged every second directly into a laptop computer.

### 2.3. Soil Sampling and Laboratory Analyses

Thirty six locations in each field were selected for soil sampling at regular intervals (∼30 m × ∼20 m). This dataset was a part of already published studies [26,27]. From each sampling location, 5–8 soil cores were collected from two depths: 0–15 cm and 15–30 cm, in a radius of 1 m from the sampling node and homogenised. A total of 144 samples from both depths were collected from both fields. Soil samples were air dried, passed through a 2-mm sieve and analysed in the laboratory for determining soil texture, electrical conductivity (EC), pH, total organic carbon (TOC) and total nitrogen (TN). Soil texture was determined using the hydrometer method [28]. Soil EC and soil pH were determined using an Eijkelkamp^®^ 18.28 Multi Parameter Analyser (Eijkelkamp, Giesbeek, The Netherlands) with EC and pH probes. Soil solutions of 1:1 (soil : de-ionised water) ratio were used to determine both EC (EC_1:1_) and pH (pH_1:1_) [28]. The TOC was determined by sulphochromic oxidation according to ISO-14235 soil quality standard. TN was determined from the sum of N-Kjeldahl, N-NO_3_^−^, N-NO_2_^−^, N-NH_3_^+^ and N-organic after UV digestion.

### 2.4. Independent Calibration and Validation Subsets

Usually, the calibration and validation measurements are taken separately. For instance, from calibration locations, the gamma-ray measurements are taken for about five minutes, whereas the measurements from the validation locations and/or from the rest of the field(s) are completed while going. The discrepancy of both methods is avoided in this study which shows the real effectiveness of the gamma-ray spectroscopy. Therefore, all gamma-ray measurements were taken real-time from both fields. The field of view of gamma-ray spectrometer allowed us to match soil samples within its sensing span. In a radius of 3 m around each soil sampling location, gamma-ray measurement points were picked up and averaged to yield a mean gamma-ray spectrum that was assigned to that soil sampling location. We assumed that this mean spectrum would be representative for the nearby (within 3 m radius) soil sampling location. In each field, soil samples from all sampling locations with associated gamma-ray measurements were randomly divided into two subsets: half for calibration and the remaining half for validation. Similarly, half of the total samples from both fields were used for calibration and the remaining half for validation for combined fields. It should be noted that a few soil sampling locations were farther than 3 m from the gamma-ray measurement points. Soil samples from those soil sampling locations were not used for calibration, but they were used for validation.

### 2.5. Spectral Data Pre-Processing

The aim of spectral pre-processing was to reduce statistical noise, to increase signal-to-noise ratio and to identify the radionuclide peaks in the measured spectra. We transformed multichannel gamma-ray data into corresponding energies using Equation (1):

(1) $$\left[E_{\gamma }(\text{MeV})=0.0117\times \gamma \right]$$

where, E_γ_ is gamma-ray energy in MeV and γ is the channel number from 1 through 256. Each channel, therefore, represents a band width of 0.0117 MeV. Gamma-ray counts in each channel were converted to count rates dividing by the life-time of a measured spectrum. A spatial filter of the moving average of seven gamma-ray sampling points was used to reduce the noise and acquire stability in gamma-ray spectra. A moving average of five energy channels was also calculated within each spectrum to further de-noise and smooth the spectra and to identify peaks. The processed spectra were used for further analysis.

### 2.6. Energy-Windows (EWs) Method

Spectral EWs of radionuclides ^40^K, ^238^U and ^232^Th (also referred to as EW_K_, EW_U_ and EW_Th_) were determined by summing the intensity of gamma-ray counts on the energy spectrum surrounding the peaks of radionuclides as suggested by [29]. Total radioactivity in terms of total counts (TC) was also used as a broad window. The EWs with their photo-peak centres are shown in Table 1.

**Figure 1.**
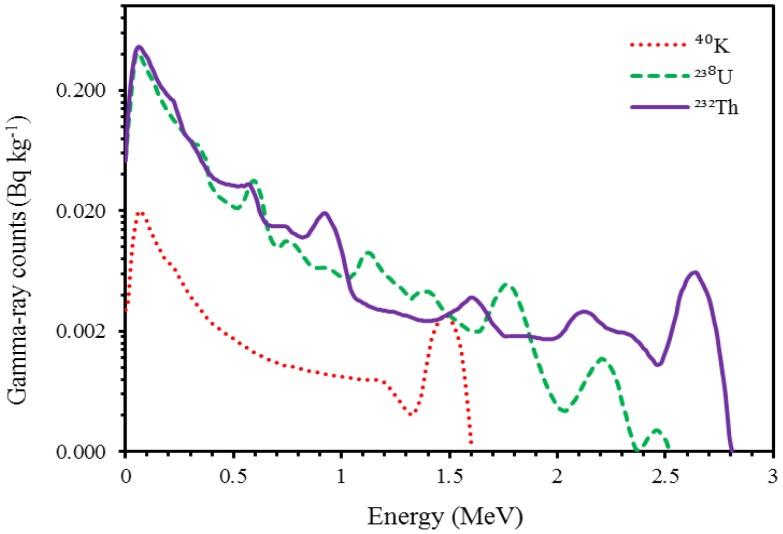
Standard spectra of ^40^K (dotted line), ^238^U (dashed line) and ^232^Th (solid line) collected by the Mole at 1 Bq·kg^−1^ activity concentration in calibration setup [24].

First, the effects of other radionuclides in a certain EW of an element were removed using sensitivity analysis [7]. Second, the count rates were converted to elemental concentrations using stripping algorithms as instructed in IAEA [7]. Stripping factors (e.g., *α*, *β* and *γ*) and sensitivities were determined from the standard spectra (a standard spectrum is the pure response of a detector system to 1 Bq·kg^−1^ source of a given radionuclide in a given geometrical setting [23] of the spectrometer (Figure 1).

### 2.7. Full-Spectrum Analysis (FSA) Method

The full-spectrum analysis (FSA) method incorporates information from nearly the entire gamma-ray spectrum. The FSA method can be based on a multivariate calibration method [22] or on a simulation theory [23]. The FSA method based on the multivariate statistics does not identify individual correlations of radionuclides with a certain soil property; rather it establishes a relationship between a soil property and the entire gamma-ray spectrum. In this study, we used the FSA method based on Monte Carlo simulations of radiation transport developed by [23]. This FSA method yields concentrations of radionuclides (*i.e.*, ^40^K, ^238^U and ^232^Th) just like the EWs methods, which makes the comparison between the two methods easy. In this method, the standard spectra of ^40^K, ^238^U and ^232^Th, with an activity concentration of 1 Bq·kg^−1^, are fitted to the measured spectrum using a Chi-square (χ^2^) algorithm [23]. Multipliers of the standard spectra of the radionuclides are thus generated that are equal to the activity concentrations of these radionuclides in Bq·kg^−1^ units.

### 2.8. Data Analysis

Exploratory bivariate analysis, based on linear regression and correlation, is a common method of data analysis to explore relationships between radiometric data and soil properties [15]. In calibration datasets, concentrations of radionuclides obtained from the EWs and the FSA methods were linearly regressed to soil properties to expose correlations between them. The developed regression models were then used to predict soil properties in the validation datasets. The strength of relationships between predicted and measured soil properties was tested with coefficient of determination (R^2^) and the root-mean squared error of prediction (RMSEP). Moreover, the statistical significance of the models was also tested using F-statistics values at 5% and 1% significance levels. Finally, we calculated the ratio of percentage deviation (RPD), which is a ratio of standard deviation of a reference soil property and its RMSEP, to test the prediction ability of models. RPD values greater than 1.4 can potentially be used for prediction of soil properties [22].

## 3. Results and Discussion

### 3.1. Study Fields and Descriptive Statistics of Soil Properties

**Figure 2.**
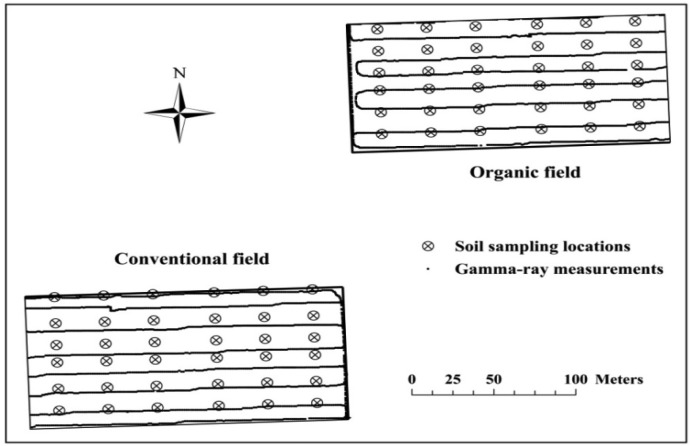
The study fields are shown with soil sampling locations (circles with crosses). The black dots (lines) are gamma-ray measurements measured every second in the field.

Locations of about 4,000 proximally sensed gamma-ray measurements and 72 sampling locations are shown in Figure 2.

Descriptive statistics of laboratory measured soil properties are given in Table 2. Soil properties showed overall narrow ranges. All soil properties of both fields showed similar statistics because both fields were located nearby. The widest range was found for sand content and the narrowest for the pH in both fields. The amount of TOC was slightly lower in the 15–30 cm soil depth in both fields because fertilisers and manures are not applied so deep.

### 3.2. Description of Gamma-Ray Spectra

**Figure 3.**
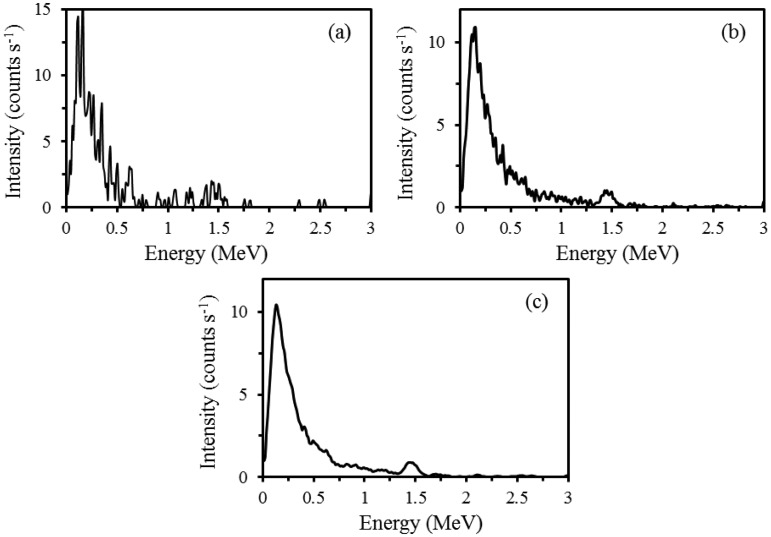
Examples of (**a**) a raw gamma-ray spectrum measured every second; (**b**) moving average of seven spatial spectra in a row and (**c**) moving average of five channels within a spectrum.

Raw gamma-ray spectra measured every second were very noisy (Figure 3a). Spatial filtering of spectra using seven point moving average removed some of the noise and reduced fluctuations between the consecutive energy channels (Figure 3b). Performing moving average of five channels within each spectrum yielded further smoother spectra and improved signal-to-noise ratio and well-shaped peaks were visible (Figure 3c). Smoothed spectra also improved correlations between radionuclides concentrations and soil properties. The amount of noise in the gamma-ray spectra is due to the sensitivity of measurement. The sensitivity of gamma-ray measurements depends on the type of detector crystal, volume of the detector and the sampling period [11]. The detector crystal and its volume are fixed and therefore longer sampling period (time) can improve the measure of certainty. To increase the sampling period, however, data acquisition speed should be reduced, so it will cost more time for scanning a field. The certainty in gamma-ray measurement is therefore a compromise between data acquisition speed and sampling period. Spatial integration of spectra rather than sampling time can also increase certainty in gamma-ray spectra.

The overall gamma-ray count rate was very low when compared with a typical airborne gamma-ray spectrum reported by [10]. Very low number of gamma-ray counts in measured spectra is probably due to a sandy loam texture of the fields with leached profile. Overall low number of counts also indicates that the soils are very young because they were reclaimed from the Ijsselmeer in the 1960s. Among the three radionuclides, only ^40^K showed a prominent peak at 1.46 MeV in the measured spectra, whereas the peaks of ^238^U and ^232^Th were very small. A small peak of ^137^Cs was also noticed around 0.66 MeV. Low number of counts in ^238^U and ^232^Th windows indicates that the soil is lacking in ferruginous materials [9], whereas a relatively high signal of ^40^K indicates that the soil is young and rich in K feldspar. Higher energy channels beyond 1.90 MeV collected either very low or zero counts as shown in Figures 3. In total radioactivity, the most part of radioactivity is contributed by the ^40^K and the other two radionuclides contribute the least.

### 3.3. Sensitivities and Stripping Factors for EWs

Sensitivities and stripping factors or ratios for EWs determined from the standard spectra of the three radionuclides are given in Tables 3 and 4. Sensitivities of radionuclides removed the effects of other radionuclides in the principal EW of a certain radionuclide, whereas the stripping ratios converted the count rates into elemental activity concentration in Bq·kg^−1^. Sensitivities and stripping factors calculated in this study were different from those determined by [7] because these parameters depend on the type of detector used and its geometry.

### 3.4. Correlation between the FSA and the EWs Concentrations

Significant linear correlations (Pearson's correlation coefficient, *r* > 0.80) were found between activity concentrations of radionuclides determined by the FSA and the EWs methods. The highest correlation was found for ^40^K (r = 0.90). High correlation between radionuclide concentrations measured by the FSA and the EWs methods indicates that accumulation of gamma rays of each radionuclide in its representative EW represents its distribution in the rest of the energy spectrum. The number of counts in the EWs, therefore, can serve as an indicator to reflect the presence of counts of a specific radionuclide in the entire gamma-ray spectrum. In the FSA method, the main contribution of a radionuclide comes from its respective EW and less from the rest of energy spectrum due to overlapping signals of all radionuclides in the continuum part of the spectrum.

### 3.5. Calibration of Radionuclide Data

Calibration results of individual and combined fields are shown in Table 5. In individual fields, the R^2^ values greater than 0.23 were statistically significant at 5% probability level (*p* = 0.05), whereas the R^2^ values greater than 0.35 were statistically significant at 1% probability level (*p* = 0.01). Similarly in combined fields, relations were significant at 5% and 1% probability level when the R^2^ values were greater than 0.13 and 0.18, respectively (Table 5). Both methods (the FSA and the EWs) established similar correlations with soil properties in the top 0–15 cm and the 15–30 cm soil depths of individual and combined fields. Overall low correlations of radionuclides with soil properties may be attributed to the low number of counts of radionuclides in this study because the number of counts of radionuclides is directly related with the strength of correlations of soil properties [15]. However, the highest number of counts were exhibited by ^40^K and much fewer by ^232^Th and ^238^U. But, most correlations were established between soil properties and ^232^Th and ^238^U radionuclides despite of yielding low number of counts (Table 5). This may be attributed to the fact that most soil properties are related with ^232^Th and ^238^U radionuclides rather than ^40^K depending on the composition of the soil.

The strength of correlations was mostly higher in the top 0–15 cm depth than the 15–30 cm depth. Similarly, the strength of correlations was higher in the individual fields than when combining them. Trends of correlations in the calibration were almost consistent across methods (FSA and EWs) but were not so consistent across fields. This means that relations between radionuclides and soil properties are site-specific and are likely to depend upon the geochemistry and internal soil processes of a soil. In the top 0–15 cm depth, the FSA method showed a good correlation for clay and pH (R^2^ ≥ 0.43) and a weak correlation for TOC and TN in individual fields (R^2^ ≥ 0.32), whereas the EWs method yielded similar correlations for these soil properties in the conventional field, but weak correlations in the organic field. Most soil properties were correlated with ^232^Th in the top 0–15 cm soil depth and with ^238^U in the 15–30 cm depth of individual fields. This implies that the signal attenuation in the 15–30 cm depth is more for ^232^Th compared with ^238^U. More signal attenuation for ^232^Th gives less accurate correlations. It may also be because ^238^U is sourced from slightly deeper in the soil profile and also possesses ability to escape the deeper soil layers than the other radionuclides. Clay showed a consistent correlation with ^232^Th in the top 0–15 cm soil depth in both fields and methods. Good correlation of clay content with radionuclides data seems direct due to its consistency, whereas correlations of other soil properties with radionuclides may be indirect because they also have good correlations with clay content. For instance, in the top 0–15 cm soil depth in the conventional field, clay is correlated with sand (R^2^ = 0.67), pH (R^2^ = 0.49) and TN (R^2^ = 0.28). A high correlation of clay content with sand is because sand is the mirror image of clay content. Similar correlations of clay were also found with other soil properties in the organic field and in the 15-30 cm soil depths. The highest correlation in combined field was noticed for sand content (R^2^ = 0.46) in the EWs method in both depths. It should be noted that correlations between radionuclide data and soil properties were variable across fields but comparatively consistent across methods.

Not all radionuclides were positively correlated with soil properties. For example, ^232^Th showed a positive correlation with clay, pH and TN. The ^232^U showed a positive correlation with sand and TOC and negative correlation with other soil properties, whereas ^40^K showed a negative correlation with TOC.

### 3.6. Prediction of Soil Properties Using the FSA Method in Individual Fields

In the conventional field, good correlations were found between measured and predicted clay, pH, TN and TOC (R^2^ ≥ 0.45) in the top 0–15 cm depth (Table 6). Contrary to the top 0–15 cm depth, generally lower correlations were found between measured and predicted soil properties in the 15–30 cm soil depth (R^2^ ≤ 0.37). The highest prediction accuracy was shown by pH (R^2^ = 0.37) in the 15–30 cm depth. In the organic field, clay, sand, pH and TN were significantly predicted (R^2^ ≥ 0.35) in the top 0–15 cm depth. The highest accuracy was found for clay content (R^2^ = 0.73). In the 15–30 cm soil depth, clay and sand were predicted with a good accuracy (R^2^ ≥ 0.52). A soil property that showed a lower correlation in the calibration was generally predicted with a lower accuracy and vice versa.

### 3.7. Prediction of Soil Properties Using the EWs Method in Individual Fields

Prediction accuracies of soil properties in the EWs method were comparable with the FSA method, however, slightly better prediction were found for a few soil properties (Table 6). In the conventional field, clay, sand, pH, TOC and TN were significantly predicted (R^2^ ≥ 0.38) in the top 0–15 cm soil depth. The highest accuracy was obtained for TN (R^2^ = 0.75). In the 15–30 cm soil depth, silt, sand and pH were predicted with fair correlations (R^2^ ≥ 0.35) and TN showed significant but a lower accuracy (R^2^ ≥ 0.26). In the organic field, clay, sand, pH, TOC and TN were predicted significantly (R^2^ ≥ 0.28) in the top 0–15 cm soil depth, whereas in the 15–30 cm depth, clay, sand and pH were significantly predicted (R^2^ ≥ 0.28). Clay showed good prediction accuracy in both depths (R^2^ ≥ 0.62). Higher prediction accuracies were found for those soil properties that showed higher correlations in the calibration.

### 3.8. Prediction of Soil Properties in Combined Fields

When both fields were combined, the accuracy of predictions of soil properties was comparable in both methods, but decreased as compared with the individual fields (Table 7). The highest prediction accuracy was found for clay content (R^2^ = 0.63) in the FSA method in the top 0–15 cm depth. All other soil properties were predicted with lower accuracies in both methods and depths (R^2^ ≤ 0.42).

### 3.9. Comparison between the FSA and the EWs Methods

Accuracies of prediction of soil properties in the FSA and EWS methods were comparable and both methods can be used to find relationships with soil properties. The EWs method, however, yielded slightly better results than the FSA method for a few soil properties, which is unexpected because the FSA method may have advantage over the EWs method. Slightly lower accuracy of predictions in the FSA method may be attributed to the uncertainties in deriving radionuclide activity concentrations caused by the covariance between the standard spectra of radionuclides, which are increased compared with the EWs method. The increased covariance is caused by the inclusion of the Compton part of the gamma-ray spectrum for ^238^U and ^232^Th, since their spectra are most similar in the continuum part [23]. This is the drawback of this FSA method. Although the FSA method is advantageous that accounts for the entire gamma-ray spectrum in gamma-ray spectroscopy, the EWs method can also establish accurate relations between radionuclides and soil properties. The FSA method used in this study is faster than the EWs method and can convert raw spectra into elemental concentrations based on the standard spectra of radionuclides. The EWs is a simple and relatively easy method when sensitivities and stripping factors of the spectrometer are known.

Better predictions were found in individual fields than when combining them. Low accuracy of predictions in combined fields is attributed to different radionuclides correlating a specific soil property in both fields during calibration. For example, in the top 0–15 cm depth for both methods, TN was best correlated with ^232^Th in the conventional field, whereas it was best correlated with ^40^K in the organic field (Table 5). Combining fields, TN yielded a lower correlation with ^40^K for both methods those results in lower prediction accuracy. Similarly, other soil properties lose their accuracy in combined fields. Results of diminished accuracies in combined fields are consistent with those of [25], who found better prediction accuracies in field-scale studies.

### 3.10. Prediction Accuracies in the Top 0–15 cm and 15–30 cm Soil Depths

Lower prediction accuracies of soil properties in the 15–30 cm depth imply that the proportion of detected gamma-ray signal decreases with increasing soil depth or thickness. Increasing bulk density further attenuates the gamma-ray signal and reduces the gamma-ray emission [8]. Signal attenuation prevents the accurate determination of soil properties from the 15–30 cm soil depth because fewer number of gamma-ray counts are escaped the soil matrix from the deeper soil layers. Attenuation of gamma rays from deeper soil depths may be even more when a low number of gamma rays, as in this study, is emitted by the soil. Low correlation of radionuclides with the soil properties of the 15–30 cm depth is also because they were not correlated with those of the top 0–15 cm depth. This is consistent with the results from [22]. Correlation between radionuclides and soil properties at 10 cm interval down to 30 cm decreases with increasing soil depth [8]. Fifty percent of the observed spectra originates from the top 10 cm soil and 90% originates from the top 30 cm. Results suggest that gamma-ray spectroscopy can generally benefit soil characterisation for annual crops where the condition of the seedbed is important because the method is restricted to near surface soil sensing. The benefits of the method can be extended to perennial crops when subsurface soil properties are correlated with the surface soil properties.

### 3.11. Prediction of Soil Properties Based on RPD Statistic

In bivariate data analysis, such as simple correlation and linear regression, the significance of models is normally tested by the R^2^, standard error of estimate or RMSEP and F-test values. In contrast, when hyper-spectral data of sensors (*i.e.*, gamma-ray data) are combined with multivariate calibration methods, the predictability or significance of models is hardly tested using F-statistics because the number of predictor variables is mostly higher than the number of observations. Therefore, the predictability of a model is tested using R^2^, RMSEP and ratio of percentage deviation (RPD) statistics [22]. In this study, we attempt to use F-statistics and RPD values to compare the models. With the new developments in statistical and mathematical techniques, the appropriate statistical parameters can be chosen for assessment of different prediction models based on the type of method used for analysis.

**Figure 4.**
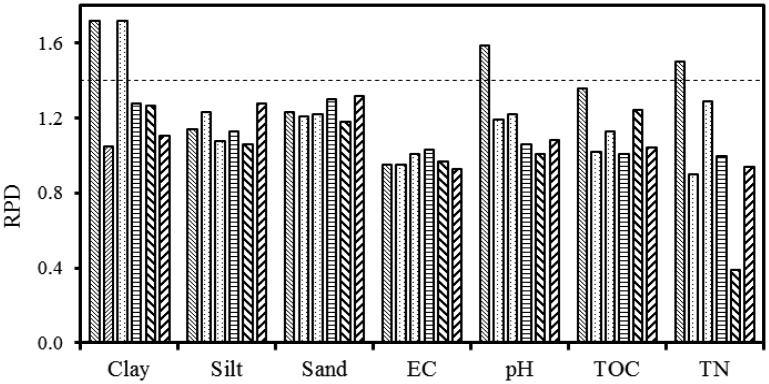
RPD statistic of individual and combined fields in 0–15 cm and 15–30 cm depths for the FSA method. Legend: 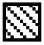 = conventional field 0–15 cm depth, 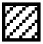 = conventional field 15–30 cm depth, 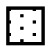 = organic field 0–15 cm depth, 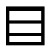 = organic field 15–30 cm depth, 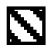 = combined fields 0–15 cm depth and 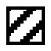 = combined fields 15–30 cm depth. Dashed line indicates the RPD value of 1.4, which is a threshold commonly used in chemometrics.

**Figure 5.**
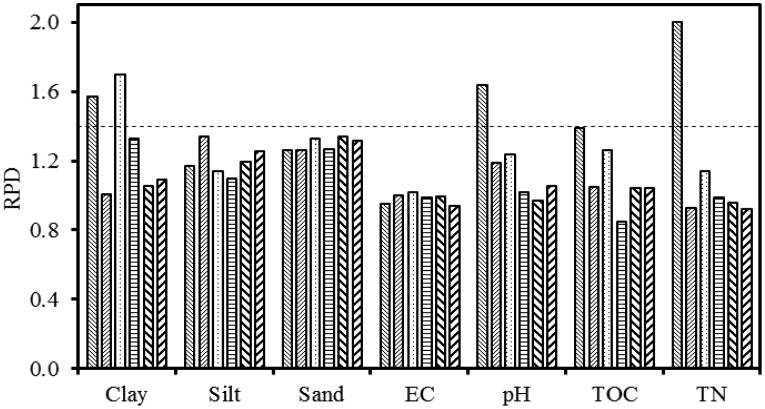
RPD statistic of individual and combined fields in 0–15 cm and 15–30 cm depths for the EWs method. Legend: 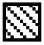 = conventional field 0–15 cm depth, 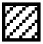 = conventional field 15–30 cm depth, 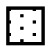 = organic field 0–15 cm depth, 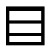 = organic field 15–30 cm depth, 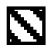 = combined fields 0–15 cm depth and 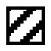 = combined fields 15–30 cm depth. Dashed line indicates the RPD value of 1.4, which is a threshold commonly used in chemometrics.

The RPD values of predicted soil properties were mostly higher in the top 0–15 cm soil depths and lower in the 15–30 cm depths for individual and combined fields (Figures 4 and 5), which are consistent with the F-statistics and R^2^ values listed in Tables 6 and 7. Clay content showed RPD values greater than 1.4 (a threshold that is widely used in chemometrics) in the 0–15 cm soil depth for both fields and methods. The highest RPD value was obtained for TN (2.0) in the EWs method in the 0–15 cm depth of the conventional field. Soil pH and TN also showed RPD > 1.4 in the conventional field for both methods. Based on the RPD values we can suggest that clay, pH and TN can be predicted successfully using gamma-ray spectroscopy when combined with either the FSA or the EWs data analysis method. Other soil properties showed lower RPD values in individual fields. When combining fields, generally lower RPD values were obtained.

### 3.12. Gamma-Ray Spectroscopy and Soil Characterisation

Results from this study indicate that relationships exist between certain soil properties and radionuclide data, which suggest a potential role of gamma-ray spectroscopy in soil property mapping. The relationship between ^232^Th and clay content in surface soil depths indicates that clay content can be measured by measuring ^232^Th signal. Correlation between ^232^Th and clay, pH and TN were consistent across fields and methods. Results of correlations between ^232^Th and clay content are consistent with those of [11,20,24,25], but are not consistent with those of [8], who correlated TC with clay content. We did not find TC significantly correlating with any soil property in any field and method. The TC can be used to relate clay content if other radionuclides also correlate with clay content. The ^40^K was least correlated with soil properties. Both the FSA and the EWs methods elucidate that clay content appears to have a direct relationship with radiometric data, whereas good correlations between radiometric data and other soil properties, such as sand, pH and TN, may be due to their correlations with clay content.

The influence of different field management systems on soil property prediction was also studied. Soil property predictions were not so consistent across fields, but the soil properties of both fields showed similar statistics. This difference may be due to the management, as in one field the fertilisers were added. Statistics of radionuclides, such as ^40^K, ^232^Th and ^238^U were similar from both fields. Adding fertilisers in the conventional field may change the activities of radionuclides if radioactive activities of radionuclides are measured from small samples just after application of fertilisers. But in this study, the emission of natural radioactivity from radionuclides was measured from bulk soil and therefore there was not much difference in the number of counts of radionuclides from both fields. There is also no evidence in literature that fertilisers can affect the gamma-ray emission from bulk soil when measured using field gamma-ray spectroscopy.

## 4. Conclusions

We demonstrated the usefulness of gamma-ray spectroscopy to predict soil properties using the EWs and the FSA methods. Radionuclide concentrations determined by the EWs and the FSA methods show good correlations with each other meaning that the number of counts of a radionuclide in its EW reflects the number of counts elsewhere in the spectrum.

Both methods yield comparable predictions when regressed against soil properties. In the conventional field, clay, pH and TN are predicted with a good accuracy in the top 0–15 cm soil depth in both methods, whereas in the organic field, this is only so with clay. The highest prediction accuracy is found for TN in the conventional field when combined with the EWs method.

Prediction accuracies of soil properties are higher in the individual fields than when combining them and thus calibration at the field level is required. Only clay content is predicted in combined fields with a good accuracy in both methods.

Good prediction accuracy for clay content in both methods and fields leads us towards the conclusion that clay content appears to have a direct relationship with radiometric data, whereas the good correlations with other soil properties, such as sand, pH and TN may be due to their correlations with clay content.

Good prediction results suggest a potential role of gamma-ray spectroscopy in modelling and mapping soil properties. Soil properties in the top 0–15 cm soil depths are predicted better than in the 15–30 cm soil depths. This implies that gamma-ray spectroscopy can generally benefit soil characterisation for annual crops where the condition of the surface layer and seedbed is important. The method is not suited for determining small differences in structure resulting from management. As for the methodology, from the findings of this study we conclude that the EWs method can establish relations between radionuclide data and soil properties as accurate as the FSA method.

## Figures and Tables

**Table 1.** Conventional energy windows (EWs) in gamma-ray spectroscopy [7].

**Radionuclide**	**Radioisotope**	**Photo-Peak Centres (Mev)**	**Energy Window (Mev)**
Potassium (^40^K)	^40^K	1.46	1.36–1.56
Uranium (^238^U)	^214^Bi	1.76	1.66–1.86
Thorium (^232^Th)	^208^Tl	2.61	2.41–2.81
Total count	-	-	0.04–2.81

**Table 2.** Descriptive statistics of soil properties of both fields (All laboratory analyses were done with the highest accuracy according to the appropriate ISO standards).

**Soil Property**	**Conventional Field (*n*= 36 for Each Depth)**				**Organic Field (*n*= 36 for Each Depth)**			
	
	**Min**	**Max**	**Mean**	**SD**	**Min**	**Max**	**Mean**	**SD**
*0–15 cm soil depth*								
Clay content (%)	16.0	22.0	18.9	1.55	17.0	22.4	19.7	1.38
Silt content (%)	15.0	25.0	19.5	2.38	9.0	24.0	14.9	3.56
Sand content (%)	53.0	68.0	61.5	3.48	56.4	73.2	65.4	4.44
EC (mS·m^−1^)	37.0	45.0	40.6	1.96	27.0	42.0	33.8	4.00
pH	7.6	7.7	7.6	0.02	7.6	7.8	7.7	0.06
TOC (mg·g^−1^)	11.3	12.7	12.0	0.37	9.4	12.8	11.4	0.92
TN (mg·g^−1^)	1.2	1.5	1.3	0.06	1.3	1.5	1.4	0.06
*15–30 cm soil depth*								
Clay content (%)	15.8	23.0	18.4	1.61	16.0	19.4	18.1	0.96
Silt content (%)	16.0	28.0	21.6	2.61	15.0	19.7	17.9	1.06
Sand content (%)	50.2	67.0	60.0	3.82	61.3	69.0	64.0	1.59
EC (mS m^−1^)	23.0	35.0	29.6	2.55	22.2	29.5	26.0	1.90
pH	7.8	8.0	7.8	0.04	7.8	7.9	7.8	0.02
TOC (mg·g^−1^)	8.6	10.9	9.9	0.57	9.2	10.3	9.7	0.22
TN (mg·g^−1^)	1.1	1.6	1.3	0.16	1.1	1.3	1.2	0.06

**Table 3.** Sensitivities (S) of EWs calculated from the standard spectra of radionuclides.

**Radionuclides**	**Sensitivities (S)**		

	**EW_1K_** ^a^	**EW_2U_** ^a^	**EW_3Th_** ^a^
K (^40^K)	0.0328	0	0
U (^238^U)	0.0605	0.0658	0.0030
Th (^232^Th)	0.0483	0.0372	0.0963

Note:

a Subscrits 1–3 indicate the number of energy windows in sequence on the gamma-ray spectrum.

**Table 4.** Stripping ratios/factors calculated from the sensitivities of the spectrometer (sensitivity values were used from Table 3 for calculating their ratios).

**Stripping Parameters**	**Ratio of Sensitivities (S)**	**Stripping Ratio**
α	S_2Th_/S_3Th_ = 0.0372/0.0963	0.3867
β	S_1Th_/S_3Th_ = 0.0483/0.0963	0.5018
γ	S_1U_/S_2U_ = 0.0605/0.0658	0.9193
a	S_3U_/S_2U_ = 0.0030/0.0605	0.0453
b	S_3K_/S_1K_ = 0/0.0328	0
g	S_2K_/S_1K_ = 0/0.0328	0

**Table 5.** Calibration statistics for individual as well as combined fields.

**Soil Property**	**Conventional Field (n = 18 for Each Depth)**				**Organic Field (n = 18 for Each Depth)**				**Combined Fields (n = 36 for Each Depth)**			
		
	**FSA**		**EWs**		**FSA**		**EWs**		**FSA**		**EWs**	
		
	**RN**^a^	**R^2^**	**RN**	**R^2^**	**RN**	**R^2^**	**RN**	**R^2^**	**RN**	**R^2^**	**RN**	**R^2^**
*0*–*15 cm soil depth*												
Clay (%)	Th	0.50	Th	0.51	Th	0.43	Th	0.60	Th	0.38	Th	0.29
Silt (%)	U	0.21	U	0.18	Th	0.14	Th	0.31	U	0.12	Th	0.35
Sand (%)	Th	0.17	U	0.18	Th	0.22	Th	0.44	Th	0.26	Th	0.46
EC (mS·m^−1^)	TC	0.16	TC	0.16	Th	0.22	Th	0.48	U	0.08	Th	0.36
pH	Th	0.47	Th	0.47	Th	0.52	Th	0.59	Th	0.20	K	0.08
TOC (mg·g^−1^)	K	0.65	K	0.50	TC	0.32	U	0.10	K&U	0.43	U	0.17
TN (mg·g^−1^)	Th	0.33	Th	0.42	K	0.33	K	0.15	K	0.13	K	0.09
*15*–*30 cm soil depth*												
Clay (%)	U	0.34	U	0.36	U	0.20	U	0.15	U	0.35	U	0.29
Silt (%)	U	0.34	U	0.36	U	0.11	U	0.11	U	0.37	U	0.39
Sand (%)	U	0.41	U	0.44	U	0.25	U	0.22	U	0.43	U	0.46
EC (mS·m^−1^)	U	0.24	U	0.10	Th	0.01	K	0.01	Th	0.02	K	0.01
pH	U	0.35	U	0.23	Th	0.05	Th	0.04	U	0.14	U	0.15
TOC (mg·g^−1^)	U	0.16	U	0.17	U	0.11	K	0.07	TC	0.01	TC	0.01
TN (mg·g^−1^)	U	0.37	U	0.25	U	0.04	Th	0.01	Th	0.00	TC	0.02

Note:

a RN stands for radionuclides, which shows the best correlation with a soil property.

**Table 6.** Statistics of validation/predictions in individual fields using the FSA and the EWs methods.

**Soil Property**	**Conventional Field (*n*= 18 for Each Depth)**						**Organic Field (*n*= 18 for Each Depth)**					
	
	**FSA**			**EWs**			**FSA**			**EWs**		
			
	**R^2^**	**RMSEP**	***p***	**R^2^**	**RMSEP**	***p***	**R^2^**	**RMSEP**	***p***	**R^2^**	**RMSEP**	***p***
*0–15 cm soil depth*												
Clay (%)	0.65	0.96	0.000	0.59	1.06	0.000	0.73	0.81	0.000	0.67	0.82	0.000
Silt (%)	0.19	2.07	0.068	0.27	2.01	0.028	0.16	3.42	0.105	0.21	3.24	0.056
Sand (%)	0.31	2.90	0.017	0.38	2.83	0.006	0.35	3.67	0.009	0.40	3.36	0.005
EC (mS·m^−1^)	0.18	2.25	0.081	0.18	2.25	0.081	0.10	3.95	0.204	0.19	3.89	0.071
pH	0.65	0.01	0.000	0.65	0.01	0.000	0.39	0.05	0.006	0.43	0.05	0.003
TOC (mg·g^−1^)	0.45	0.27	0.002	0.47	0.27	0.002	0.17	0.78	0.090	0.34	0.69	0.011
TN (mg·g^−1^)	0.56	0.04	0.000	0.75	0.03	0.000	0.41	0.05	0.004	0.28	0.05	0.024
*15–30 cm soil depth*												
Clay (%)	0.13	1.52	0.134	0.11	1.58	0.186	0.55	0.57	0.000	0.62	0.55	0.000
Silt (%)	0.30	2.33	0.020	0.42	2.13	0.004	0.22	0.91	0.051	0.18	0.93	0.079
Sand (%)	0.28	3.34	0.023	0.35	3.18	0.010	0.52	1.09	0.001	0.51	1.12	0.001
EC (mS·m^−1^)	0.05	2.90	0.396	0.11	2.74	0.177	0.04	1.94	0.403	0.05	2.01	0.360
pH	0.37	0.04	0.007	0.41	0.04	0.004	0.34	0.02	0.012	0.28	0.02	0.025
TOC (mg·g^−1^)	0.03	0.56	0.463	0.07	0.54	0.294	0.09	0.17	0.240	0.08	0.20	0.247
TN (mg·g^−1^)	0.26	0.15	0.032	0.26	0.14	0.030	0.11	0.06	0.169	0.15	0.06	0.110

**Table 7.** Statistics of validation/predictions for combined fields using the FSA and the EWs methods.

**Properties**	**0–15 cm Soil Depth (n = 36)**						**15–30 cm Soil Depth (n = 36)**					

	**FSA**			**EWs**			**FSA**			**EWs**		

	**R^2^**	**RMSEP**	**p**	**R^2^**	**RMSEP**	**p**	**R^2^**	**RMSEP**	**p**	**R^2^**	**RMSEP**	**p**
Clay (%)	0.63	1.00	0.000	0.40	1.25	0.000	0.18	1.13	0.009	0.19	1.17	0.007
Silt (%)	0.06	3.67	0.145	0.16	3.60	0.014	0.29	2.29	0.001	0.27	2.39	0.001
Sand (%)	0.25	3.77	0.002	0.33	3.61	0.000	0.33	2.84	0.000	0.31	3.01	0.000
EC (mS·m^−1^)	0.13	3.95	0.028	0.15	4.24	0.019	0.06	2.90	0.156	0.05	2.82	0.184
pH	0.24	0.05	0.003	0.08	0.05	0.080	0.14	0.03	0.027	0.15	0.03	0.019
TOC (mg·g^−1^)	0.42	0.54	0.000	0.32	0.59	0.000	0.08	0.43	0.096	0.08	0.43	0.096
TN (mg·g^−1^)	0.30	0.06	0.001	0.19	0.06	0.008	0.11	0.12	0.053	0.04	0.12	0.224
